# Detour Ahead: Incretin Hormone Signaling Alters Its Intracellular Path as β-Cell Failure Progresses During Diabetes

**DOI:** 10.3389/fendo.2021.665345

**Published:** 2021-04-15

**Authors:** Mehboob A. Hussain, Erinn Laimon-Thomson, Syed M. Mustafa, Alexander Deck, Banya Song

**Affiliations:** ^1^ Department of Internal Medicine, Division of Metabolism, Endocrinology and Diabetes, University of Michigan, Ann Arbor, MI, United States; ^2^ College of Literature, Science and Arts, University of Michigan, Ann Arbor, MI, United States

**Keywords:** diabetes, beta cell dysfunction, KATP channel, GPCR coupling, incretin hormone

## Introduction

The early events leading to β-cell failure during the pathogenesis of type 2 diabetes mellitus (T2DM) remain poorly understood (1). Upon glucose stimulation, insulin is released from β-cells in a biphasic manner with an initial first peak of insulin release (first phase), which – if the glucose stimulus persists - is followed by a second prolonged phase of insulin release (2–5). Defective first-phase insulin release is among the earliest markers that predict the development of T2DM (2–4). This defect persists in patients with T2DM and is also found in first-degree relatives of patients with T2DM (4). However, the molecular underpinnings of this particular defect have largely remained elusive. A recent report by Odouri et al. (6) now provides important new insight that may help understand the early stages in the development of β-cell dysfunction of T2DM. In this commentary we place these important findings (6) in the broader context of incretin hormone signaling and the early defects that occur in incretin action in β-cells during diabetes pathogenesis. We close by outlining new avenues for scientific inquiry, which the findings by Oudori et al. have opened with their observations.

## Stimulus-Secretion Coupling and Amplification of Insulin Release

### Gαs or Gαq Signaling-Dependent Mechanisms

Glucose-stimulated insulin secretion (GSIS) occurs through a cascade of precisely orchestrated electro-physiologic events in pancreatic β-cells (7, 8). Glucose transport into β-cells and enhanced glucose metabolism increase the cellular ATP/ADP ratio, leading to closing of ATP-sensitive K^+^ (K_ATP_) channels. Restricted K^+^ exit through K_ATP_ channels depolarizes the β-cell plasma membrane, which in turn is followed by activation and opening of voltage dependent Ca^2+^ channels (VDCC). Calcium influx and thus increased cytoplasmic Ca^2+^ concentration in the β-cell, triggers insulin vesicle exocytosis (9–13).

GSIS is amplified by hormones and neurotransmitters, of which many function by activation of G-protein coupled receptors *via* trimeric G-proteins containing Gαs or Gαq, which signal, respectively, *via* cAMP and phospholipase C (PLC)-diacylglycerol (DAG)/-inositol 1, 4, 5-triposphate (IP_3_) (9, 14). Although both Gαs-cAMP and Gαq-PLC-DAG)/IP_3_ are clearly recognized to amplify β-cell GSIS, the exact mechanisms of their signaling pathways, how they differ and where they overlap remain incompletely understood. Both Gαs-cAMP (via PKA-CREB activation) and Gαq-DAG-protein kinase C (PKC; *via* ERK1/2) activation increase IRS2 levels in β-cells, which is a central mediator in maintaining expression of PDX1 and other transcription factors that form a gene-regulatory network to maintain β-cell identity and maturity (15, 16). Gαs-cAMP and Gαq-PLC-dependent signaling pathways both regulate inositol tri-phosphate (IP_3_) receptor activity to mobilize calcium stored in the endoplasmic reticulum (ER) into the cytoplasm; and both PKA and PKC phosphorylate select SNARE (=soluble N-ethylmaleimide sensitive factor attachment protein receptor) complex proteins (i.e. SNAP25, MUNC18) to promote insulin vesicle exocytosis (17, 18). Thus, Gαs-PKA and Gαq-DAG-PKC signaling pathways converge on common targets that regulate GSIS amplification. Nevertheless, these two signaling systems fulfill distinct and non-redundant functions as β-cell-selective ablation of either Gαs (19) or Gαq (20) results in defective β-cell function or survival. 

## The Central Role for Incretin Hormones in Regulating Insulin Secretion

Critical for preventing postprandial hyperglycemia are the amplifying effects of the incretin hormones glucagon-like peptide-1 (GLP-1) and glucose dependent insulinotropic peptide (GIP). Under physiologic conditions, the actions of these two incretin hormones are responsible for approximately 50% of insulin secretion after meal intake (21–23)

GLP-1 and GIP are released from enteroendocrine L- and K-cells, respectively, upon nutrient stimulus in the intestine. Through the circulation they reach the pancreatic β-cells, where by activating their cognate G-protein coupled receptors, they potentiate glucose stimulated insulin release (21). The receptors for both GLP-1 (GLP-1R) and GIP (GIPR)—both abundantly expressed in β-cells, belong to the class B (secretin family) G-protein coupled receptors (GPCR); and both couple primarily to the G-protein Gαs (21). Consequently, in the case of both hormones, binding and activation of their respective receptors stimulates intracellular cAMP synthesis and downstream signaling that is mediated by protein kinase A (PKA) and by the guanyl nucleotide exchange factor exchange protein activated by cAMP 2A (EPAC2A) (24). Cyclic AMP–PKA activation, *via* phosphorylation of PKA targets, stimulates multiple pathways within the β-cell that promote β-cell proliferation, survival, changes in gene expression as well as GSIS amplification. These are extensively reviewed elsewhere to which we refer the reader (21, 25) EPAC2A in β-cells, upon activation by cAMP translocates to the cytoplasmic membrane where it regulates exocytosis and mobilize intracellular calcium, thereby also amplifying GSIS (24, 26).

In this context, is important to point out that in intestinal L-cells GLP-1 is produced through differential processing of proglucagon. More recently, there is increasing experimental evidence that GLP-1 may not only be produced in intestinal L-cells but also through differential posttranslational processing of proglucagon in pancreatic endocrine α-cells, where proglucagon is primarily processed to glucagon (27, 28). As such, GLP-1 produced in α-cells may also act on β-cells in a paracrine manner (27, 28). In certain experimental conditions, paracrine α−to β cell signaling of proglucagon products appears to be critical for normal β-cell function Furthermore, glucagon—the principal proglucagon-derived product in α-cells—binds to and activates both the glucagon receptor (GCGR) and GLP-1R (albeit with markedly reduced affinity as compared to GLP-1) on β-cells (29–32). Like the incretin hormone receptors, GCGR belongs to the class B secretin family of GPCRs, but couples to both Gαs and Gαq to stimulate, respectively cAMP- and DAG)/(IP_3_)-dependent signaling (33–35). GCGR–dependent signaling has been primarily described in hepatocytes (33–35) and - absent clinching experimental data - it remains unclear whether GCGR normally also signals *via* both Gαs and Gαq in β-cells.

Importantly, the loss of incretin hormone action in amplifying GSIS is an early characteristic of T2DM (23, 36–38). In individuals with defective first-phase insulin release and future risk of developing frank T2DM, pharmacologic GLP-1 receptor agonist treatment restores first-phase insulin secretion (39). However, quite early after the discovery of GLP-1 and GIP a remarkable difference was observed in their actions on β-cells of individuals with T2DM (22, 38). While treatment with GLP-1 potently amplifies GSIS in patients with T2DM, GIP treatment fails to do so; despite the fact that receptors of both of these hormones couple to Gαs to stimulate intracellular cAMP-dependent signaling. As a consequence of these observations, GLP-1 receptor agonists have quickly become a focus of scientific inquiry as well as of drug development for T2DM. Although clearly a key characteristic of β-cell failure in T2DM, the difference in action between GLP-1 and GIP in islets of T2DM has at the molecular level remained thus far unexplained. 

## Linking Defective K_ATP_ Channel Activity and Defective Incretin Hormone Action In β Cells

The carefully conducted studies reported by Odouri et al. (6) provide a potential molecular explanation for the differences in GLP-1R and GIPR signaling in healthy β-cells versus those in T2DM. The initial focus of Odouri et al. was on the role of K_ATP_ channels in β-cells (6, 40, 41). Partial loss of K_ATP_ channels increases electrical excitability and insulin secretion, resulting in hyperinsulinemia in humans and in mice (42, 43). Complete loss of K_ATP_ channels causes permanent depolarization and chronically elevated intracellular Ca^2+^ concentrations at all glucose levels. However paradoxically and through unknown mechanisms, the absence of K_ATP_ channels results in a down-regulation of insulin secretion and defective GSIS (42, 43).

Mice generated by Odouri et al. lack the pore-forming Kir6.2 component of the K_ATP_ channel specifically in β-cells (β-Δ*Kcnj11* mice), and confirming prior observations are actually glucose intolerant (6, 42, 43). Importantly however, GLP-1 effects on amplifying GSIS was partially retained while the effects of GIP were practically absent (6). These observations are remarkably similar to findings in humans who develop T2DM (see above). 

### Signaling Detour for GLP-1R but Not for GIPR

A key observation by Odouri et al. is that in these “hyper-excited” K_ATP_ channel-defective mouse islets, cAMP synthesis through GLP-1R or GIPR activation is markedly diminished. Importantly, while GIPR-dependent signaling is as a consequence practically silenced, the GLP-1R switches coupling from Gαs to Gαq, thereby engaging an alternate signaling pathway and allowing GLP-1 to potentiate GSIS in β-cell (6). In contrast GIPR fails to switch its coupling from Gαs to Gαq. Importantly, Odouri et al. show that GLP-1R does not couple through Gαq in healthy control islets, but only in those with down-regulated K_ATP_ channel activity (6).

In additional studies Odouri et al. examined the KK-Ay mouse, which spontaneously develops a human T2DM-like phenotype. While β-cells from non-diabetic control KK mice showed normal electro-physiologic activity and patterns of GSIS, β-cells from KK-Ay mice were chronically depolarized and lacked GSIS. Treatment with GLP-1 but not with GIP amplified GSIS in KK-Ay islets. And GLP-1 effects were inhibited with the Gαq antagonist YM-254890 (It would help had the authors also examined the effects of the more specific Gαq antagonist FR900359) (6). Thus, as in T2DM, in KK-Ay mice, β-cell GLP-1R but not GIPR signals *via* Gαq while Gas-dependent signaling becomes defective.

The potential importance of these findings to understand β-cell failure in a broader context of T2DM comes with additional studies, in which Odouri et al. (6) expose both mouse and human islets to elevated glucose levels (as found in T2DM) for 3 to 5 days—a maneuver that causes chronic K_ATP_ channel closure (6, 44). Remarkably, when such treated islets were returned to lower (i.e. normoglycemic) glucose levels, their K_ATP_ channels remained suppressed and GSIS remained defective. However, similar to the observations made in β-Δ*Kcnj11* mice, GLP-1–induced GSIS potentiation was maintained, whereas GIP treatment had no such effect (6). And again, GLP-1 receptor signaling had switched from Gαs to Gαq coupling, whereas GIPR signaling had not made that switch.

These important observations by Odouri et al. provide a molecular explanation for the long known “incretin bias” in T2DM and link this particular phenomenon to a β-cell autonomous change in K_ATP_ channel activity (6, 44)—for which the underlying pathogenic mechanisms remain incompletely understood.

While the observations made by Odouri et al. provide experimental evidence that chronic (3–5 days) exposure to elevated glucose levels can lead to a down-regulation of K_ATP_ channel activity, it remains unclear whether other influences to which the β-cell is exposed in early T2DM pathogenesis (e.g., altered circulating lipid profiles, low level inflammation and likely additional as yet unrecognized factors) that precede hyperglycemia may also cause a down-regulation in K_ATP_ channel activity.

## Discussion

As all good science, the studies reported by Oduori et al. (6) have not only significantly advanced the field but also point in which direction to look further. The remarkable finding that in β-cells GLP-1R coupling switches during diabetes pathogenesis adds impetus to understand in more detail the mechanism and pathways of Gαs- and Gαq-dependent signaling in β-cell. Odouri et al. findings raise many questions related to incretin biology and to β-cell (dys-)function in T2DM pathogenesis as well as questions related to the treatment of β-cell dysfunction. Among these will undoubtedly be the following:

1. Are there circumstances in which a switch in GLP-1R coupling from Gαs to Gαq is of physiologic importance (as opposed to pathologic, i.e., diabetes mellitus). The larger questions is whether the switch in GLP-1R coupling serves a particular physiologic purpose in the β-cell or whether this switch is a only manifestation of dysfunction and disease.
2. What are the intracellular mechanisms that underlie the switch from for Gαs to Gαq at the GLP-1R receptor? Insight into the molecular underpinnings of the switch from Gαs to Gαq would greatly enhance our understanding of changes in β-cell function early in the pathogenesis of diabetes mellitus.
3. How does Gαq-mediated signaling lead to improved β-cell function, and how does it differ from Gαs-mediated signaling in modulating β-cell function? Gαs- and Gαq-dependent signaling pathways converge at multiple levels. But their distinct roles remain poorly understood.
4. How do GLP-1R-Gαs “uncoupling” and GLP-1R-Gαq coupling modify β-cell proliferation survival, and maintenance of β-cell maturity? This question is an extension of the preceding question.
5. How does the glucagon receptor fare in the rochade from Gαs to Gαq coupling through GPCRs in β-cells? In T2DM, does glucagon receptor-dependent signaling also switch away from Gαs and signal primarily *via* Gαq? Glucagon uniquely activates both GLP-1R and GCGR (whereas GLP-1 does not activate GCGR. GCGR thus functions akin to an incretin hormone. Whether and how GCGR signaling changes during diabetes is unknown. The role of glucagon in β-cell (dys-) function remains to be fully understood.
6. How do circulating lipids, circulating and local islet cytokines, intracellular alterations such as organelle (ER, mitochondria, Golgi apparatus) stress interplay with the silencing of Gαs-coupling of incretin receptors and switch from Gαs to Gαq signaling? Odouri et al. show that hyperglycemia causes a switch in GLP-1R coupling. Whether other participants in T2DM pathogenesis also promote this switch remains unclear.
7. Which therapeutic maneuvers will allow GLP-1R signaling of the failing β-cell to couple back with Gαs and also reactivate GIPR signaling in β-cells?

The following three questions pertain to therapeutic approaches of β-cell dysfunction in light of the new findings by Odouri et al.

1. Would GLP-1R agonists that bias coupling through Gαq rather than Gαs be more effective in treating β-cell failure?
2. Will pharmacologic targeting of primarily Gαq-coupled GPCR, such as muscarinic M3 acetylcholine receptors (15, 45, 46) be more effective than GLP-1R agonists in preventing or reversing β-cell failure?
3. Would simultaneous stimulation of Gαs- and Gαq-dependent signaling pathways in promoting β-cell function and survival be superior to stimulation of either pathway alone?

While this article was under peer-review, a newly published study indicates a role for GIP-GIPR in amplifying amino-acid-induced glucagon secretion from islet alpha-cells as an additional mechanism of incretin hormone action (47).

## Author Contributions

All authors contributed to the article and approved the submitted version.

## Funding

Supported in part by NIH DK126011, AG071232, DK020572.

## Conflict of Interest

The authors declare that the research was conducted in the absence of any commercial or financial relationships that could be construed as a potential conflict of interest.
